# An analysis of occupational exposure to particulate matter and erythrocyte morphological abnormalities among charcoal-grill restaurant workers

**DOI:** 10.3389/fpubh.2026.1953388

**Published:** 2026-09-15

**Authors:** Burcu Yuksel, Serap Arsal Yildirim

**Affiliations:** Kocaeli Vocational School of Health Services, Kocaeli University, Kocaeli, Türkiye

**Keywords:** erythrocyte morphology, grill restaurant workers, indoor air quality, occupational exposure, particulate matter, poikilocytosis

## Abstract

**Background:**

Workers in charcoal- and wood-fired grill restaurants are exposed to high concentrations of combustion-derived particulate matter (PM) and carbon monoxide during long shifts in poorly ventilated kitchens. Whether this exposure is associated with measurable changes in circulating erythrocyte morphology is not established.

**Methods:**

This study used a comparative cross-sectional design and compared charcoal-grill workers (*n* = 25) with a group of office personnel (*n* = 25) in Kocaeli University. At each occupational unit, the researchers assessed indoor air conditions, measuring particulate matter (PM) mass concentration, carbon monoxide (CO), carbon dioxide (CO₂), ambient temperature, and relative humidity. Peripheral blood smears were processed for a subgroup of the subjects (restaurant *n* = 25; office *n* = 19). For everyone, we examined and averaged the outcomes from two distinct 1,000-cell samples to quantify 10 structural anomalies in erythrocytes. We compared groups using Benjamini-Hochberg-adjusted Mann–Whitney U tests and applied negative binomial regression for anomaly counts, adjusting for age, sex, and education.

**Results:**

Restaurant worksites had markedly higher PM concentrations (median 117.8 vs. 0.23 μg/m^3^), CO and CO₂ than offices (all *p* < 0.01). Restaurant workers reported more wheezing (12/25 vs. 0/25) and dyspnoea (11/25 vs. 1/25, both FDR-corrected *p* < 0.05). In unadjusted analyses, total anomalies were higher in restaurant workers, but no single abnormality survived correction. After adjustment for age, sex and education, four specific poikilocytes remained significantly elevated: dacrocytes (adjusted IRR 2.46, 95% CI 1.36–4.44), elliptocytes (IRR 2.33, 1.16–4.71), sickle-form cells (IRR 4.47, 1.41–14.17) and schistocytes (IRR 1.56, 1.03–2.35). The crude difference in total anomaly burden did not survive confounder adjustment (IRR 1.57, 0.65–3.78).

**Conclusion:**

Grill restaurant work involves high-particulate exposure associated with stress-related erythrocyte poikilocytes, suggesting mechanical and oxidative membrane damage rather than generalised morphological changes. However, due to subclinical anomaly rates, demographic confounders, and missing control data, these findings remain hypothesis-generating. Because of the limited sample size and the investigative nature of this research, the current findings should be interpreted as tentative and primarily useful for formulating future hypotheses. Furthermore, its cross-sectional design and lack of direct measurements of oxidative stress preclude establishing causality or verifying biological pathways.

## 1 Introduction

Charcoal- and wood-fired grilling is a defining feature of the Turkish restaurant sector, and the workers who operate these grills spend long shifts within a metre of the combustion source. The fumes they inhale are chemically complex: charcoal combustion releases carbon monoxide, carbon dioxide, nitrogen oxides, fine and ultrafine respirable particulate matter, a range of volatile organic compounds, and particle-bound polycyclic aromatic hydrocarbons and trace metals (1). In a survey of 14 charcoal grill restaurants in Kocaeli, the same setting studied here, Taner et al. (2) found that charcoal combustion was the dominant source of indoor fine particulate matter, with a mean PM₂.₅ concentration of 84.8 μg/m^3^ (range 21.6–254.8 μg/m^3^), and that toxic elements such as arsenic, chromium, selenium, and vanadium were strongly enriched in the fine fraction. High particulate and gas concentrations have also been measured directly in restaurant kitchens elsewhere, including in New York (3) and China (4). Although the World Health Organisation’s 2021 guideline does not define a threshold specific to the respirable fraction, its closest comparable benchmark, PM₂.₅, sets an annual mean limit of 5 μg/m^3^ and a 24-h mean limit of 15 μg/m^3^ (5); since the respirable fraction also encompasses PM₂.₅, these values provide a conservative point of comparison. The mean respirable PM concentration measured in the restaurant settings of the present study (191.8 μg/m^3^) markedly exceeds both this guideline and the occupational levels reported above. Grill workers are therefore among the more heavily exposed occupational groups in the food service sector.

The health consequences of this exposure have been documented across several occupational groups. Biomonitoring studies of grill and kitchen workers have shown elevated urinary levels of hydroxylated PAH metabolites on working days compared with non-working days, confirming a genuine internal dose rather than mere ambient exposure (6). Occupational exposure to cooking fumes has also been linked to oxidative and genotoxic effects: in a controlled study of Chinese restaurant kitchen workers, malondialdehyde, 8-hydroxy-2-deoxyguanosine, and micronucleus frequency were higher in exposed workers than in controls, and these differences persisted after adjustment for age, body mass index, smoking, and alcohol use (7). At the population level, long-term particulate exposure is associated with subclinical inflammation and altered haematological and thrombotic markers (8). Comparable occupational biomonitoring in other dust- and fume-exposed trades, such as the cytogenetic changes documented in the buccal mucosa of dental laboratory technicians, illustrates how accessible cellular biomarkers can reveal the systemic footprint of workplace exposure (9). Respiratory effects follow the same pattern: in a cross-sectional study of 224 workers across 142 restaurants in Thailand, restaurant workers reported chronic respiratory symptoms at twice the rate or more compared with a control group (10), and cooking-fume-exposed kitchen workers in Iran showed acute, partly reversible decrements in pulmonary function relative to an unexposed control group (11).

What has received far less attention is the effect of this exposure on the morphology of circulating red blood cells. Erythrocytes are unusually vulnerable to oxidative injury because their membrane is rich in polyunsaturated fatty acids, they are continuously exposed to high oxygen tension, and the autooxidation of haemoglobin generates a steady internal flux of reactive oxygen species (12). Inhaled particulates provide an additional external oxidative load. Seaton and colleagues were the first to show that particulate air pollution leads to changes in blood parameters, proposing this as a mechanism underlying its cardiovascular effects (13). *In vitro*, urban particulate matter has been shown to adhere to and be internalised by erythrocytes, raising intracellular reactive oxygen species, depleting glutathione, oxidising haemoglobin, and altering cell shape (14). The downstream biomechanical consequence is a loss of membrane deformability and stability, which single-cell and rheological studies attribute directly to oxidative reorganisation of the membrane cytoskeleton (12, 15). In controlled human inhalation experiments, fine particulate exposure has been shown to increase oxidative stress markers on circulating red blood cells and to disturb erythrocyte turnover (16). Indoor PM exposure has been separately linked to reductions in anaemia-related blood indices, such as mean corpuscular volume and mean corpuscular haemoglobin (17).

These membrane changes have a recognisable morphological signature. Oxidative and mechanical stress on the erythrocyte membrane shifts the population of cell shapes towards poikilocytes: teardrop-shaped dacrocytes and fragmented schistocytes are classic markers of mechanical membrane injury, while elliptocytosis reflects reduced membrane stability, and burr cells, spherocytes, and target cells reflect other patterns of membrane and cytoskeletal disruption. Because these forms can be counted directly on a stained peripheral smear, erythrocyte morphology offers an inexpensive and widely accessible index of the systemic burden of inhaled exposure, one that does not require specialised biochemical assays. We have previously applied this peripheral-smear approach to another dust-exposed occupational group, podologists, among whom erythrocyte anomaly counts were higher than in controls (18). To our knowledge, however, no study has examined whether the peripheral smear of charcoal grill workers shows this signature.

The present study addresses that gap. We characterised the indoor air quality in charcoal-grill restaurants (restaurant workers, *n* = 25). We compared it with that observed in office environments (office workers, *n* = 25) for respirable PM, CO, CO₂, humidity, and temperature. We also examined whether erythrocyte morphological abnormalities and respiratory symptoms differed between workers in these two settings. Because a single worksite air measurement cannot be assigned to an individual worker, we treated the two environments as a high-exposure vs. low-exposure contrast rather than attempting an individual dose–response analysis. We asked whether restaurant work is associated with a distinct erythrocyte morphological profile after accounting for demographic differences between the groups. We hypothesised that workers in this high-particulate environment would show a higher frequency of erythrocyte morphological abnormalities than low-exposure office workers, and that any excess would fall preferentially on poikilocytes associated with oxidative and mechanical membrane stress, namely dacrocytes, schistocytes and elliptocytes, rather than on forms reflecting nutritional or maturation disturbance. All 10 abnormalities routinely identifiable on a peripheral smear were therefore scored, so that both the specificity and the presence of any effect could be assessed.

## 2 Materials and methods

### 2.1 Study design and setting

This comparative cross-sectional investigation was carried out in Kocaeli, which is a highly industrialised region in northwestern Türkiye recognised for the widespread use of charcoal grilling. The research assessed the differences between two distinct professional cohorts: personnel employed in wood- and charcoal-fired grill eateries, which constituted a setting with elevated combustion emissions, and administrative staff, who served as a baseline with minimal exposure. Previous studies has frequently used university students and administrative personnel as reference demographics for similar occupational hazard evaluations [e.g., (19, 20)]. Following this common practice, the control group was made up of staff holding office jobs in the secretariat, clerical, and student affairs divisions of Kocaeli University. The data collection process entailed monitoring the indoor atmospheric quality at the employment sites, administering a standardised survey to the participants, and evaluating peripheral blood smears.

### 2.2 Participants

The exposed group comprised 25 workers recruited from grill restaurants serving charcoal-cooked meat, offal and wing dishes. The comparison group comprised 25 workers from office environments with no occupational exposure to combustion emissions. Participants were included if they had worked in their current setting for a period sufficient to reflect habitual occupational exposure and provided informed consent. All 50 participants completed the questionnaire. Peripheral blood smears suitable for morphological scoring were obtained from all 25 restaurant workers and 19 of the 25 office workers; smears from the remaining 6 controls were unavailable for analysis, and these participants were therefore excluded from the erythrocyte comparison but retained for the demographic and symptom analyses. Administrative office staff were selected as the control group because they live in the same industrial metropolitan area, have similar socioeconomic backgrounds, and have similar access to healthcare to the grill employees. However, they all carry out their duties in an indoor settings free from occupational cooking emissions and combustion byproducts. As a result, they operate as a practical, low-exposure baseline for the targeted pollutants, despite the two groups not matching on educational attainment and gender, as detailed further below. Viable peripheral blood smears were collected from all 25 restaurant employees, whereas only 19 of the 25 administrative subjects yielded readable slides. The remaining six reference individuals lacked smears suitable for morphological evaluation due to either technical flaws in slide preparation or insufficient specimen volume. While these six individuals were still included in evaluations derived from the survey data, they were omitted from the comparative study of red blood cell morphology. Participants were recruited consecutively from participating workplaces. Eligible participants were adults currently employed in the respective setting; individuals with a known haematological disorder or a condition expected to alter erythrocyte morphology were not eligible for the blood-smear analysis. All participants in the exposed group served exclusively as a grill operator, meaning their primary station throughout their shift was immediately next to the wood- or charcoal-fired grills. By deliberately excluding general restaurant personnel, such as waitstaff or food-preparation crews, this selection criterion ensured that the study group consisted solely of the occupational role most directly and continuously exposed to combustion fumes.

### 2.3 Questionnaire

A structured questionnaire recorded age, sex, educational level, occupational tenure in years, daily shift length in hours, and habitual use of masks and gloves at work. Respiratory and allergic history was recorded using the European Community Respiratory Health Survey (ECRHS) questionnaire (21), noting the presence or absence of wheezing, dyspnoea, audible wheeze, chest tightness, nocturnal dyspnoea, cough, asthma attacks, physician-diagnosed asthma, respiratory medication use, rhinitis, eczema, and a family history of atopic or respiratory disease. The survey also documented relevant individual and familial medical histories about the study’s endpoints, including doctor-diagnosed asthma, asthma exacerbations, ongoing use of respiratory drugs, allergic rhinitis, eczema, and any familial history of respiratory or atopic conditions. However, the instrument did not account for other potential confounders. These unrecorded factors included tobacco use, alcohol intake, anaemia and other haematological conditions, long-term illnesses, recent infectious episodes, nutritional status, and prior workplace hazards. The possible impact of disregarding these variables is addressed in the Limitations section. The ECRHS respiratory questionnaire is a widely used and validated instrument for assessing respiratory symptoms in adult populations (21).

### 2.4 Indoor air quality assessment

Indoor air quality was characterised at each worksite during normal operating hours. Respirable particulate matter (PM₄, particulate matter with an aerodynamic diameter less than 4 μm) was determined according to NIOSH Method 0600 (Particulates Not Otherwise Regulated, Respirable) (22), adapted for area rather than personal sampling. A calibrated cyclone sampler connected to an SKC air sampling pump, with the draw speed set to 2.2 L/min (NIOSH-0600; 1998), was mounted on a tripod stand as close as possible to the employees’ respiratory tracts, and sampling continued even when the employees temporarily left the room to preserve a continuous area-level exposure estimate. The pump system was calibrated at the Kocaeli OHSI (Occupational Health and Safety Research and Development Institute) Regional Laboratory. The sampler separated the respirable particulate fraction, with a median cut point of approximately 4 μm, onto a pre-weighed 25-mm glass microfibre filter (Cat#: IW-AE2500). Filters were equilibrated for at least 2 h in a temperature- (20 ± 1 °C) and humidity-controlled (50% ± 5% relative humidity) environment both before and after sampling, then weighed on an analytical balance with 0.001 mg sensitivity; the respirable PM mass concentration was calculated as the captured mass divided by the sampled air volume and expressed in micrograms per cubic metre. The method’s working range is approximately 0.5–10 mg/m^3^, as defined in the NIOSH Manual of Analytical Methods. Using a factory-calibrated multi-parameter device (Testo 440), we simultaneously logged gas-phase metrics specifically carbon monoxide (CO), carbon dioxide (CO₂), ambient temperature, relative humidity, and air velocity. Environmental PM collection and gas tracking occurred concurrently during a single working shift at each location, with multiple gas measurements recorded to document brief environmental fluctuations. Assessments spanned 12 distinct restaurant locations and four administrative spaces. For respirable PM, 17 filter evaluations were carried out across the 12 eateries (with multiple tests at certain venues) and four within the offices; this lower frequency of office sampling was justified by the stable, minimal pollutant concentrations found there. Importantly, data collection relied heavily on stationary, environmental monitors rather than individual exposure tracking. Consequently, no personnel wore personal dosimeters, nor were breathing zone or biological measurements taken. Thus, the recorded data represent the facility’s general ambient pollutant levels and serve only as an approximation of the actual inhaled dose for each specific employee, which naturally fluctuates based on their duties, mobility, and time spent in the area. Instead of consolidating multiple gas readings into a single average per location, each measurement was retained as a distinct data point. In Table 1, the overall indoor air quality statistics for each cohort were calculated using the median and interquartile range of these aggregated readings. This procedure accounts for the discrepancy between the higher number of gas-phase data points (38 for restaurants, 6 for offices) and the lower number of respirable PM data points (17 for restaurants, 4 for offices), as the latter relied on a single gravimetric filter per assessment. Conversely, during the supplementary internal cohort evaluation detailed in Section 3.5, individual employees were linked to the environmental data recorded at their exact workstation.

**Table 1:** Indoor air quality parameters by environment, median (IQR).

Parameter	Restaurant	Office	*p*
PM (μg/m^3^)	117.8 (51.0–313.2)	0.23 (0.19–0.51)	<0.001
CO (ppm)	2.0 (0.8–4.8)	0.0 (0.0–0.0)	<0.001
CO₂ (ppm)	679 (537–983)	292 (286–295)	<0.001
Temperature (°C)	34.2 (30.1–38.3)	27.0 (25.1–27.7)	0.010
Relative humidity (%)	30.6 (26.2–36.0)	47.5 (45.8–51.5)	0.003

IQR, interquartile range; PM, respirable particulate matter; CO, carbon monoxide; CO_2_, carbon dioxide. Values are medians (IQR) across all indoor air quality readings within each environment (12 independent restaurant sites and 4 office sites were sampled; respirable PM: 17 filter samples across the 12 restaurant sites and 4 office samples; gas-phase parameters: 38 restaurant and 6 office readings); *p* values from Mann–Whitney U tests.

The electrochemical carbon monoxide sensor (operating between 0–500 ppm with a 0.1 ppm resolution) recorded 0.0 ppm at the administrative sites. This shows that concentrations fell below the device’s detection threshold, rather than an absolute absence of CO. Because these unmeasurable levels were used only for descriptive group overviews and preliminary internal correlations rather than being incorporated into dose–response modelling, we retained the raw instrument output of 0.0 ppm without applying substitution or data imputation. Ultimately, the administrative cohort serves as a minimal-exposure benchmark; therefore, the handling of these undetected values does not compromise the comparative analysis between the two groups. Indeed, values at or below the detection limit in the control setting merely emphasise the marked disparity in exposure between the restaurants and offices.

### 2.5 Peripheral blood smear analysis

Peripheral venous blood was collected from each participant, and thin blood films were prepared and stained using the May-Grünwald-Giemsa method. For each participant, two independent films were prepared and examined under oil immersion with a light microscope. 1,000 erythrocytes were assessed on each film, yielding 2000 cells per participant. Ten erythrocyte morphological abnormalities were enumerated: microcytes, macrocytes, dacrocytes, elliptocytes, target cells, sickle-form cells, ovalocytes, schistocytes, keratocytes and Howell-Jolly bodies. Each abnormality was expressed as a count per 1,000 erythrocytes. Agreement between the two films was assessed by Pearson correlation of the total abnormality count and was high in both groups (*r* = 0.82 in restaurant workers and *r* = 0.95 in office workers), supporting the reliability of the counts; the two films were therefore averaged to yield a single value per participant per abnormality. Blood specimens were collected midway through the employees’ daily shifts. This timing strategy was intentionally selected to capture the profile of circulating red blood cells amid ongoing workplace exposure, rather than evaluating them before or after their working hours.

### 2.6 Statistical analysis

Analyses were performed in Python (version 3) using the scipy and statsmodels libraries. Continuous variables are summarised as median and interquartile range and were compared between groups using the Mann–Whitney U test, with Cliff’s delta reported as a non-parametric effect size. Categorical variables were compared using the chi-squared test or the Fisher exact test, as appropriate to the expected cell counts. For the 10 erythrocyte abnormalities and, separately, for the respiratory symptoms, *p*-values were adjusted for multiple comparisons using the Benjamini-Hochberg false discovery rate procedure. The primary evaluations comparing the restaurant and office groups were conducted individually, using each subject’s personal symptom reports and red blood cell data. Because environmental air quality metrics were not included as predictive variables in these initial tests, the statistical comparisons remain unaffected by any clustering of employees within specific venues. This clustering effect is relevant only to the secondary internal group evaluation detailed in Section 3.5, in which multiple restaurant employees shared identical worksite-level exposure metrics. Given that the 25 grill workers were spread across 12 distinct sampled locations, with most sites contributing only a single participant, a standard multilevel (random-intercept) model would lack sufficient statistical power to estimate variance across worksites accurately. Consequently, rather than implementing a mathematically unstable hierarchical model, we present the internal group findings as preliminary Spearman rank correlations, while acknowledging this clustering phenomenon.

Because the exposed and comparison groups differed in demographic composition, each erythrocyte abnormality count was additionally modelled using negative binomial regression with restaurant work as the exposure of interest and age, sex and educational level as covariates. The negative binomial distribution was selected to accommodate overdispersion in the count outcomes, and the dispersion parameter was chosen for each outcome by minimising the Akaike information criterion. Results of these models are reported as adjusted incidence rate ratios with 95% confidence intervals. A two-sided *p*-value below 0.05 was considered statistically significant. Considering the limited participant size, we approached this investigation as an exploratory analysis and implemented multiple procedures to verify the robustness of our statistical models. We detected significant overdispersion across all count variables (with Poisson Pearson chi-square/df ratios ranging from 2.4 to 7.5), which adequately justified using a negative binomial framework rather than a standard Poisson model. To ensure that individual outliers were not unduly influencing the results, the model-derived confidence intervals for every statistically significant anomaly were validated against a non-parametric bootstrap utilising 2,000 participant resamples. The robust alignment between these two methods confirmed the reliability of the estimates. Throughout our reporting, we provide exact confidence intervals and base our interpretations on the steadiness of these diagnostic checks rather than relying exclusively on rigid significance cutoffs.

To account for potential residual confounding, we conducted an additional sensitivity analysis by incorporating occupational tenure and daily shift duration as supplementary covariates when refitting the models for the four significant anomalies. Specifically, the frequency of each abnormality was analysed using a negative binomial generalised linear model featuring a log link function, structured as:

$$\begin{array}{l} \log(E[\text{count}])=\beta_{0}+\beta_{1}(\text{restaurant})\\ +\beta_{2}(\mathrm{age})+\beta_{3}(\mathrm{sex})+\beta_{4}(\text{education}) \end{array}$$

Consequently, the exponentiated coefficient for the restaurant variable, ( $\beta_{1}$ ), represents the incidence rate ratio comparing restaurant personnel with administrative staff while holding the other covariates constant. We determined the coefficients using maximum likelihood estimation and selected the dispersion parameter that yielded the optimal fit according to the Akaike information criterion (AIC). When formally compared to a Poisson model utilising identical predictors, the negative binomial approach consistently produced a vastly superior (lower) AIC for every count variable (for instance, 284 compared to 444 for elliptocytes, and 265 vs. 336 for dacryocytes). Furthermore, its residual deviance per degree of freedom was near unity (ranging from 1.1 to 1.4), indicating an appropriate model fit with no residual overdispersion. While we evaluated less complex methodologies, such as an unadjusted Poisson model or the conversion of outcomes to binary variables, we ultimately discarded them. The Poisson approach fails to account for the prominent overdispersion, and categorising continuous data wastes valuable information. Therefore, we advance the negative binomial models, supported by the bootstrap and diagnostic validations, while continuing to characterise the entire analytical process as exploratory.

### 2.7 Ethics

All participants provided informed consent before data collection, and the study was conducted in accordance with the principles of the Declaration of Helsinki.

## 3 Results

### 3.1 Participant characteristics

The two groups were similar in age (restaurant median 42.0, IQR 32–51 years; office 39.0, IQR 35–46 years; *p* = 0.45) and occupational tenure (17 vs. 16 years; *p* = 0.53) but differed in several respects. Restaurant workers worked longer daily shifts (median 10 vs. 8 h, *p* < 0.001), were predominantly women (21/25 vs. 12/25; chi-squared *p* = 0.017) and had lower educational attainment (2/25 university-educated vs. 21/25; *p* < 0.001). Regular mask use was infrequent among restaurant workers (5/25) and absent in the office group. These imbalances are treated as confounders in the adjusted analyses (Table 2). Table 2 presents an overview of foundational participant characteristics, encompassing gender, educational attainment, employment duration, daily shift hours, and the utilisation of personal protective equipment. Reflecting the distinct physical requirements of their respective professions, 100% of the culinary personnel wore gloves, whereas none of the administrative staff did. Furthermore, clinical diagnoses of asthma, the routine use of respiratory medications, allergic rhinitis, eczema, and any documented familial predisposition to atopic or pulmonary conditions proved rare, with no substantial changes detected between the two primary groups. Additionally, an evaluation was carried out to compare the six administrative subjects who lacked a viable blood smear against the 19 controls retained for the morphological analysis. While these two subgroups were comparable in daily work hours, occupational tenure, gender distribution, and educational background, the excluded individuals were significantly older (median age of 46.5 years vs. 37.0 years; *p* = 0.006). Finally, self-reported respiratory complaints were infrequent and showed no significant differences between the two administrative subsets.

**Table 2:** Demographic and occupational characteristics of the two worker groups.

Characteristic	Restaurant (*n* = 25)	Office (*n* = 25)	*p*
Age, years, median (IQR)	42 (32–51)	39 (35–46)	0.45
Sex, women / men	4/21	12/13	0.017
Occupational tenure, years (IQR)	17 (6–30)	16 (8–20)	0.53
Daily shift length, h (IQR)	10 (10–12)	8 (6–8)	<0.001
University education, n	2	21	<0.001
Regular mask use, n	5	0	n/a

IQR, interquartile range; n, number of participants; n/a, not applicable. *p* values from Mann–Whitney U (continuous) or chi-squared/Fisher exact (categorical) tests.

### 3.2 Indoor air quality

Restaurant worksites were characterised by substantially degraded air quality relative to offices. Particulate matter concentration was two to three orders of magnitude higher (median 117.8 μg/m^3^, IQR 51.0–313.2, range 45.5–561.4, mean 191.8 μg/m^3^ vs. 0.23 μg/m^3^, IQR 0.19–0.51 in offices; *p* < 0.001); the mean exceeds the median, consistent with the right-skewed distribution indicated by the wide range. Carbon monoxide (median 2.0 vs. 0.0 ppm; *p* < 0.001), carbon dioxide (679 vs. 292 ppm; *p* < 0.001) and temperature (34.2 vs. 27.0 °C; *p* = 0.010) were all higher in restaurants, while relative humidity was lower (30.6% vs. 47.5%; *p* = 0.003; Table 1; Figure 1).

**Figure 1:**
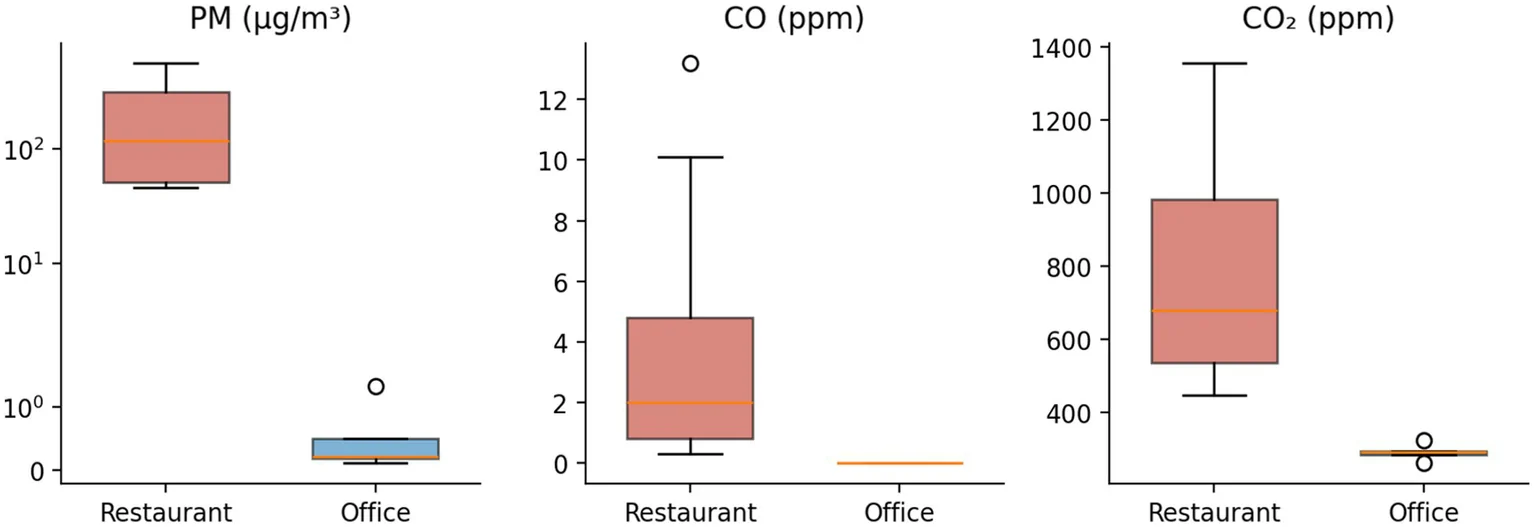
Distribution of particulate matter, carbon monoxide and carbon dioxide across restaurant and office worksites. The PM axis is on a symmetric log scale to accommodate the very large differences between environments.

### 3.3 Respiratory symptoms

Restaurant workers reported respiratory symptoms more frequently than office workers (Table 3). Wheezing (12/25 vs. 0/25; FDR *p* = 0.001), dyspnoea (11/25 vs. 1/25; FDR *p* = 0.012) and audible wheeze (7/25 vs. 0/25; FDR *p* = 0.039) were significantly more common. Chest tightness and nocturnal dyspnoea showed the same direction of effect but did not survive correction. Physician-diagnosed asthma and regular use of respiratory medication were absent in both groups. Non-parametric bootstrap sampling produced confidence intervals that were almost indistinguishable from the model-derived intervals across all four red blood cell anomalies (e.g., dacryocytes: 1.43–4.42; schistocytes: 1.03–2.69), which supports the robustness and stability of these metrics despite the restricted sample size. When daily shift duration and occupational tenure were added as covariates, the association with dacryocytes remained statistically significant (IRR = 2.41, 95% CI: 1.19–4.89, *p* = 0.015). By contrast, the effect estimates for elliptocytes, sickle-form cells, and schistocytes were attenuated and no longer reached significance. Interpreting this loss of significance requires caution, primarily because daily shift length is highly collinear with group assignment (restaurant personnel inherently worked longer daily hours) and because a six-covariate model produces considerable over-parameterisation for a sample of only 44 subjects. Consequently, we retained age, sex, and educational attainment as our core adjustment set, presenting the expanded specification solely for sensitivity analysis.

**Table 3:** Respiratory symptom prevalence by group, n reporting the symptom out of 25.

Symptom	Restaurant	Office	*p* (FDR)	Risk difference, % (95% CI)
Wheezing	12	0	0.001	+48 (26 to 67)
Dyspnoea	11	1	0.012	+40 (17 to 59)
Audible wheeze	7	0	0.039	+28 (9 to 48)
Chest tightness	5	0	0.120	+20 (3 to 39)
Nocturnal dyspnoea	5	0	0.120	+20 (3 to 39)
Cough	3	0	0.469	+12 (−3 to 30)
Rhinitis	1	0	1.000	+4 (−10 to 20)
Eczema	1	2	1.000	-4 (−21 to 13)

*p* values are from Fisher exact tests with Benjamini-Hochberg correction. Values are the number of participants (out of 25 per group) reporting each symptom. FDR, Benjamini-Hochberg false discovery rate. *P* values from Fisher exact tests. Risk differences (restaurant minus office prevalence) are shown with 95% confidence intervals calculated using the Newcombe method.

### 3.4 Erythrocyte morphology

Unadjusted comparisons of the 10 erythrocyte abnormalities are shown in Table 4. Restaurant workers had a higher total anomaly burden (median 59.5 vs. 31.0 per 1,000 erythrocytes; *p* = 0.036, uncorrected) and higher counts of microcytes, dacrocytes, and sickle-form cells (each *p* < 0.05, uncorrected). After FDR correction across the 10 abnormalities, no unadjusted comparison remained significant at the 0.05 level. This is what would be expected given the modest sample size and the demographic imbalance between the groups, which the subsequent adjusted analysis was designed to address.

**Table 4:** Erythrocyte morphological abnormalities per 1,000 erythrocytes by group, median (IQR).

Abnormality	Restaurant	Office	*p*	p (FDR)
Microcyte	5.5 (2.0–7.0)	2.0 (1.2–4.2)	0.014	0.057
Macrocyte	6.0 (4.0–10.0)	4.5 (2.5–10.0)	0.265	0.410
Dacrocyte	8.5 (5.0–10.5)	4.5 (2.8–6.2)	0.012	0.057
Elliptocyte	8.0 (5.0–12.5)	6.0 (2.5–6.8)	0.070	0.136
Target cell	0.0 (0.0–0.0)	0.0 (0.0–0.2)	0.298	0.410
Sickle-form cell	2.0 (0.0–5.5)	0.5 (0.0–1.0)	0.016	0.057
Ovalocyte	7.0 (4.0–10.5)	5.5 (3.8–11.5)	0.972	0.972
Schistocyte	8.0 (4.5–11.0)	7.0 (5.0–8.5)	0.348	0.426
Keratocyte	6.5 (4.5–9.5)	4.5 (2.5–8.2)	0.447	0.492
Howell-Jolly body	0.0 (0.0–0.0)	0.0 (0.0–0.0)	0.074	0.136
Total anomalies	59.5 (43.0–65.5)	31.0 (29.5–62.0)	0.036	0.099

*p* values are from Mann–Whitney U tests with Benjamini-Hochberg correction (p FDR). Values are counts per 1,000 erythrocytes, expressed as median (IQR); each participant’s value is the mean of two independent 1,000-cell preparations. “Total anomalies” is the sum of the 10 abnormality counts per 1,000 erythrocytes. IQR, interquartile range; FDR, Benjamini-Hochberg false discovery rate.

Because the groups differed markedly in sex and education, we modelled each abnormality using negative binomial regression, adjusting for age, sex, and educational level (Table 5). After adjustment, four abnormalities remained significantly elevated in restaurant workers: dacrocytes (IRR 2.46, 95% CI 1.36–4.44), elliptocytes (IRR 2.33, 1.16–4.71), sickle-form cells (IRR 4.47, 1.41–14.17) and schistocytes (IRR 1.56, 1.03–2.35). In contrast, macrocytes, ovalocytes, keratocytes and Howell-Jolly bodies showed no association, and the total anomaly count was not significantly different after adjustment (IRR 1.57, 0.65–3.78). The excess was therefore confined to poikilocytes classically associated with mechanical fragmentation and membrane stress rather than to a generalised rise in all abnormal forms (Figure 2). Non-parametric bootstrap resampling produced confidence intervals virtually indistinguishable from the model-derived intervals across all four red blood cell anomalies (e.g., dacryocytes: 1.43–4.42; schistocytes: 1.03–2.69), confirming the robustness and stability of these metrics despite the restricted sample size. When daily shift duration and occupational tenure were added as covariates, the association with dacryocytes remained statistically significant (IRR = 2.41, 95% CI: 1.19–4.89, *p* = 0.015). By contrast, the effect estimates for elliptocytes, sickle-form cells, and schistocytes were attenuated and no longer reached significance. Interpreting this loss of significance requires caution, primarily because daily shift length is highly collinear with group assignment (restaurant personnel inherently worked longer daily hours) and because a six-covariate model introduces considerable over-parameterisation for a sample of only 44 subjects. Consequently, we retained age, sex, and educational attainment as our core adjustment set, presenting the expanded specification solely for sensitivity analysis.

**Table 5:** Confounder-adjusted association between restaurant work and each erythrocyte abnormality (negative binomial regression, adjusted for age, sex and education).

Abnormality	Adjusted IRR	95% CI	*p*
Dacrocyte	2.46	1.36–4.44	0.003
Sickle-form cell	4.47	1.41–14.17	0.011
Elliptocyte	2.33	1.16–4.71	0.018
Schistocyte	1.56	1.03–2.35	0.034
Microcyte	1.50	0.79–2.85	0.211
Macrocyte	1.04	0.57–1.87	0.906
Ovalocyte	0.91	0.46–1.82	0.792
Keratocyte	1.02	0.50–2.07	0.954
Total anomalies	1.57	0.65–3.78	0.315

IRR, incidence rate ratio from negative binomial regression adjusted for age, sex and education; CI, confidence interval. An IRR above 1 indicates a higher count in restaurant than office workers. “Total anomalies” is the summed count of all 10 abnormalities per 1,000 erythrocytes.

**Figure 2:**
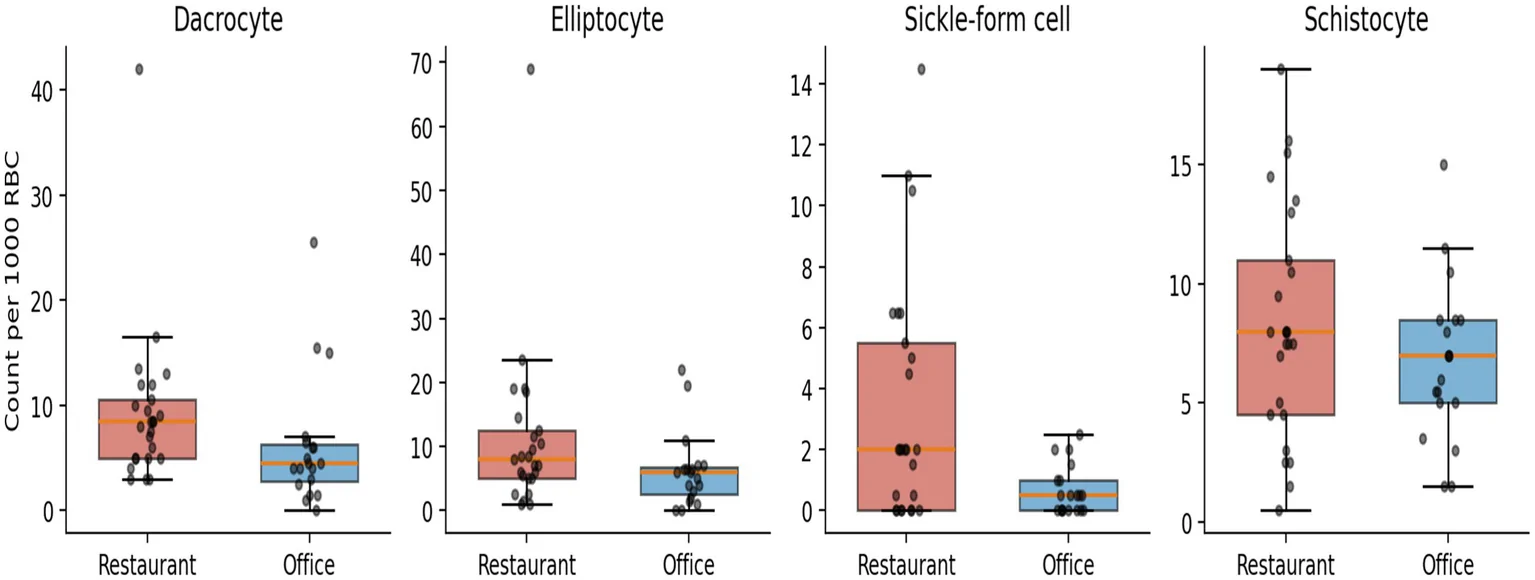
Counts per 1,000 erythrocytes for the four abnormalities that remained significantly elevated in restaurant workers after adjustment for age, sex and education. Points are individual participants; boxes show median and interquartile range.

### 3.5 Within-group exposure correlates among restaurant workers

As a supplementary analysis restricted to the 25 restaurant workers, each worker’s assigned worksite-level PM, CO, and CO₂ readings, together with daily shift duration, were correlated with the respiratory symptom score and total erythrocyte anomaly count (Spearman’s rho). Carbon monoxide concentration was significantly correlated with symptom score (rho = 0.45, *p* = 0.023), while its relationship with erythrocyte anomaly count showed the same positive direction but did not reach significance (rho = 0.33, *p* = 0.104). Particulate matter concentration showed weaker, non-significant associations with both outcomes (symptom score: rho = 0.30, *p* = 0.152; erythrocyte anomaly count: rho = 0.23, *p* = 0.272). Carbon dioxide concentration, which more plausibly indexes local ventilation and occupant density than combustion-derived toxicant load per se, was not associated with either outcome (both *p* > 0.10). Cumulative daily shift length was independently correlated with total erythrocyte anomaly count (rho = 0.43, *p* = 0.032). Duration of exposure, rather than instantaneous concentration alone, may therefore be an important determinant of the haematological response. Respiratory symptom score and erythrocyte anomaly count were themselves uncorrelated with one another (rho = −0.03, *p* = 0.894), consistent with these two outcome domains capturing at least partially distinct biological responses to the same occupational environment. Air flow rate at the worksite, which varied over a narrow range (0.05–0.60 m/s, median 0.25 m/s), was not associated with either outcome (symptom score: rho = 0.09, *p* = 0.682; erythrocyte anomaly count: rho = −0.07, *p* = 0.736). Regular mask use, reported by only 5 of the 25 workers, was associated with a non-significant trend towards lower symptom scores (median 0 among mask users vs. 2 among non-users; Mann–Whitney *p* = 0.174; point-biserial *r* = −0.30) but showed no relationship with erythrocyte anomaly count (median 127 vs. 126 per 2000 cells examined, that is across both smear preparations; *p* = 0.812; Table 6).

**Table 6:** Spearman correlations and group comparisons between indoor air exposure indices, air flow rate, shift duration, mask use, respiratory symptoms and erythrocyte anomaly burden among restaurant workers (*n* = 25).

Variable pair	Level (n)	rho	*p*
CO ↔ Respiratory symptom score	Individual (n = 25)	0.45	**0.023**
PM concentration ↔ Respiratory symptom score	Individual (n = 25)	0.30	0.152
CO₂ ↔ Respiratory symptom score	Individual (n = 25)	0.30	0.145
Shift duration ↔ Erythrocyte anomaly burden	Individual (n = 25)	0.43	**0.032**
PM concentration ↔ Erythrocyte anomaly burden	Individual (n = 25)	0.23	0.272
CO ↔ Erythrocyte anomaly burden	Individual (n = 25)	0.33	0.104
Air flow rate ↔ Respiratory symptom score	Individual (n = 25)	0.09	0.682
Air flow rate ↔ Erythrocyte anomaly burden	Individual (n = 25)	−0.07	0.736
Mask use ↔ Respiratory symptom score	Yes (n = 5) vs. No (n = 20)	–	0.174
Mask use ↔ Erythrocyte anomaly burden	Yes (n = 5) vs. No (n = 20)	–	0.812

rho, Spearman correlation coefficient; n, number of participants. Erythrocyte anomaly burden is the total count of morphological abnormalities; correlations are exploratory and restricted to the 25 restaurant workers. Mask use is a binary variable; the comparisons shown were tested with the Mann–Whitney U test rather than Spearman correlation (point-biserial *r* = −0.30 for the symptom score comparison, *r* = 0.19 for the erythrocyte anomaly comparison). Bold values denote significant results.

## 4 Discussion

Within the framework of this comparative cross-sectional desing, staff at charcoal-grill eateries worked in settings where particulate matter levels exceeded those in administrative offices by two to three orders of magnitude, accompanied by increased ambient heat and elevated concentrations of carbon monoxide and carbon dioxide. Because air conditions were evaluated on a facility-wide basis rather than through individualised exposure tracking, these environmental disparities cannot be directly linked to the specific haematological or symptomatic profiles of a single employee, aside from the secondary internal cohort evaluation discussed further on. Consequently, our primary findings reflect overarching disparities between the two cohorts: specifically, a higher frequency of self-reported respiratory complaints and a marked overabundance of irregularly shaped red blood cells (erythrocyte poikilocytes) in the culinary group. Following statistical corrections for demographic differences, the rates of dacryocytes, elliptocytes, sickle-form cells, and schistocytes remained significantly elevated among the restaurant staff, whereas the prevalence of macrocytes, ovalocytes, and other morphological variants showed no substantial divergence between the populations. The particulate concentrations we recorded are consistent with earlier characterisation of the same restaurant setting in Kocaeli, where charcoal combustion was the dominant source of indoor fine particulate matter and toxic trace elements were strongly enriched in the respirable fraction (2).

The environmental contrast between the two settings was substantial. Respirable PM concentrations were markedly higher in restaurants than in offices (median 117.8 vs. 0.23 μg/m^3^, a difference of roughly two to three orders of magnitude), a disparity like that documented in other charcoal- and gas-fired commercial kitchens (2, 23). Carbon monoxide, essentially undetectable in offices (median 0.0 ppm), reached a median of 2.0 ppm in restaurants. This is modest in absolute terms relative to levels recorded in some other restaurant kitchens (23) or in acute occupational CO incidents (24), but consistent with continuous low-level combustion-derived exposure rather than an isolated leak. Even at such modest concentrations, CO is not inert: it binds haemoglobin with roughly 200–250 times the affinity of oxygen, forming carboxyhaemoglobin and competitively impairing oxygen delivery, and it also exerts direct cellular toxicity by binding to myoglobin and cytochrome c oxidase (25). Thus, cumulative low-level exposure across a working shift could plausibly contribute to the systemic oxidative burden alongside PM. Carbon dioxide followed a smaller but still substantial gradient, with a median of 679 ppm in restaurants compared with 292 ppm in offices, approximately 2.3 times higher, and an interquartile range (537–983 ppm) that approached the 1,000 ppm threshold conventionally used as an indicator of inadequate ventilation (26). Because indoor CO₂ levels primarily reflect the balance between occupant respiration and fresh-air exchange rather than combustion emissions per se, this pattern suggests that the restaurant kitchens in this setting were not only more heavily polluted but also less well-ventilated than the office comparison sites, compounding rather than diluting workers’ cumulative exposure to combustion by-products.

The morphological pattern has a plausible biological basis. Schistocytes are fragments produced by extrinsic mechanical damage to red cells in circulation, dacrocytes arise when erythrocytes are distorted under mechanical stress, and elliptocytosis reflects reduced membrane stability (27). A selective rise in precisely these fragmentation-type and membrane-stability forms, rather than a uniform increase across all abnormalities, is more consistent with an oxidative and mechanical insult to the red cell membrane than with a nutritional or maturation disturbance, which would instead be expected to shift macrocyte and target-cell counts. Two of the four affected forms, dacrocytes and sickle-form cells, were already elevated in the unadjusted comparison, whereas elliptocytes and schistocytes reached significance only after adjustment. This is consistent with confounding by demographic differences between the groups masking a real association in the crude analysis, rather than the reverse, since the adjusted models remove variance attributable to age, sex, and education. This interpretation aligns with experimental evidence that particulate matter induces oxidative stress in erythrocytes. *In vitro*, urban particulate matter raises intracellular reactive oxygen species, depletes glutathione, oxidises haemoglobin, and alters red cell shape (14); controlled inhalation increases oxidative stress markers on circulating red cells (16); and single-cell studies show that oxidative injury reorganises the membrane cytoskeleton and reduces deformability (12, 15). The observation that the crude difference in total anomaly burden did not survive adjustment, while the mechanistically specific forms did, argues against a purely artefactual explanation driven by the demographic imbalance between the groups. It is important to stress, however, that this proposed biological mechanism is deduced from observed trends in our data and previous experimental research, rather than definitively proven in this study. We did not directly quantify specific oxidative pathways, and the cross-sectional design of our study precludes establishing a clear causal direction between occupational exposure and structural alterations in erythrocytes. Consequently, while our morphological results align closely with existing scientific literature, this agreement should be interpreted merely as supportive, not conclusive, evidence for oxidative and mechanical damage to the cellular membrane.

These findings sit within a broader literature linking cooking-fume and combustion-derived exposure to systemic effects in exposed workers. Kitchen workers exposed to cooking-oil fumes show elevated oxidative and genotoxic markers that persist after adjustment for age, body mass index, smoking and alcohol (7); grill workers show a genuine internal dose of polycyclic aromatic hydrocarbons during working days (6); and long-term particulate exposure is associated with subclinical inflammatory and haematological change at the population level (8). Our results extend this literature beyond biochemical and inflammatory markers to the morphology of circulating red cells and suggest that the peripheral smear may offer a low-cost, widely accessible index of the systemic burden of grill-smoke exposure in a setting where more specialised assays are rarely available. They are also consistent with our earlier finding of elevated erythrocyte anomaly counts, including dacrocytes, elliptocytes, and ovalocytes, among podologists exposed to inhaled nail dust (18). A shared poikilocytic response may therefore accompany occupational inhalation of diverse particulate types.

Several features of the data call for a cautious reading of the magnitude of these changes. The abnormal forms were counted per 1,000 erythrocytes, corresponding to frequencies well below 1%, and are therefore far below the thresholds at which poikilocytes such as schistocytes carry clinical significance in the diagnosis of thrombotic microangiopathy (27). The findings should thus be understood as a subclinical shift in the distribution of red cell shapes, not as evidence of a haemolytic or microangiopathic disorder. Morphological enumeration of fragmented and distorted cells is also known to be subject to observer variability, and standardised criteria exist precisely because of this subjectivity (27). The direction and specificity of the between-group difference are more informative here than the absolute counts.

From an occupational health standpoint, the combination of very high particulate concentrations, minimal ventilation, and negligible respiratory protection identifies charcoal-grill restaurants as settings in which relatively straightforward measures, such as improved local exhaust ventilation and the consistent use of respiratory protection, could plausibly reduce exposure. Reviews of charcoal-grilling emissions reach a similar conclusion, noting that engineering controls, better fuel quality and adequate ventilation are the principal available mitigation strategies (1).

To probe this relationship further, and in partial response to the individual-level limitation noted above, we conducted a supplementary within-group analysis restricted to the 25 restaurant workers, correlating each worker’s assigned worksite-level PM, CO and CO₂ readings and daily shift duration with respiratory symptom score and total erythrocyte anomaly count (Table 6). Carbon monoxide concentration and cumulative shift duration were each significantly correlated with their respective outcomes, whereas particulate matter and carbon dioxide showed only weaker, non-significant associations. Respiratory symptom score and erythrocyte anomaly count were uncorrelated with one another, consistent with these two outcome domains capturing at least partially distinct biological responses to the same occupational environment.

These within-group associations are broadly consistent with the wider literature: carbon monoxide and cumulative duration of kitchen exposure have both previously been linked to respiratory symptom burden in restaurant and kitchen workers (10, 11), and particulate matter exposure has been shown experimentally to disrupt erythrocyte turnover and alter anaemia-related blood indices via an oxidative mechanism (16, 17). They should nonetheless be interpreted with caution: because PM, CO and CO₂ were measured at the worksite rather than at the individual level, workers in the same restaurant necessarily share the same exposure value, introducing pseudo-replication that a sample of only 12 independent restaurant sites, several represented by a single worker, could not fully resolve. The weaker, non-significant relationship observed for particulate matter in a sample of this size should not be read as evidence against the mechanistic pathway described above, but rather as evidence of insufficient power to detect it.

### 4.1 Limitations

Several limitations require that these results be read as hypothesis-generating rather than confirmatory. First, the design is cross-sectional and cannot establish causation or the temporal sequence of exposure and morphological change. Second, air quality was characterised at the worksite level and could not be linked to individual workers, so the primary restaurant-vs.-office comparison was necessarily analysed as a between-environment contrast rather than as an individual dose–response relationship; the small number of office particulate measurements further limits the precision of the environmental comparison. A supplementary within-group analysis (Section 3.5, Table 6) partially addressed this limitation by correlating each restaurant worker’s assigned worksite-level readings with individual symptom and erythrocyte outcomes; because workers sharing the same restaurant necessarily shared the same exposure value, however, these correlations remain subject to a degree of pseudo-replication that a sample of only 12 independent restaurant sites, several represented by a single worker, could not fully resolve. Third, the exposed and comparison groups differed substantially in sex and educational composition. However, we adjusted for these variables; residual confounding cannot be excluded, and the strong sex imbalance in particular warrants caution. Fourth, peripheral smears were available for only 19 of the 25 controls, reducing the power of the morphological comparison and introducing the possibility of selection effects in the control blood sample. Fifth, we did not measure biochemical markers of oxidative stress, such as malondialdehyde or glutathione, that would directly corroborate the proposed mechanism, and we did not record smoking status, which is a recognised source of erythrocyte oxidative stress and a potential confounder. Finally, the sample size is modest, and the wide confidence intervals for the rarer abnormalities, notably sickle-form cells, indicate that these specific estimates are imprecise. Taken together, the small sample, the number of outcomes examined and the width of several confidence intervals mean that this is a small, exploratory study; the individual associations, particularly those resting on borderline or unadjusted *p* values, should be treated as preliminary signals to be confirmed in larger samples rather than as definitive effects. More broadly, because the exposed and comparison groups differed in sex, education and working hours, and because these factors can independently influence lifestyle, healthcare access and haematological status, residual confounding cannot be excluded and the observed differences should not be attributed to particulate exposure alone; the consistency of the dacrocyte finding across adjustment sets is reassuring, but the other associations in particular require confirmation in matched or larger samples. A further and important limitation is that several established determinants of respiratory symptoms, oxidative stress and erythrocyte morphology were not recorded. Chief among these is smoking status, a recognised source of erythrocyte oxidative stress and poikilocytosis and a plausible confounder given that smoking prevalence may differ by occupation; we also lacked data on alcohol consumption, anaemia and other blood disorders, chronic disease, recent infection, nutritional status and previous occupational exposures. Because these variables were uncontrolled, they may explain part of the observed associations, and this uncertainty applies to all the reported comparisons. In addition, exposure was assessed only through fixed-location area monitoring, which is a proxy for personal inhaled dose. Without personal sampling or biomarkers of internal dose, we cannot quantify the exposure received by each worker, and this exposure misclassification is likely to be non-differential. The exclusion of these six controls was not entirely random, since they were older than the controls retained for morphological analysis; because older age could itself be associated with erythrocyte morphology, their omission could, in principle, have biased the control distribution. Two considerations temper this concern: age was included as a covariate in all adjusted models, and the excluded controls did not differ from the retained controls in sex, education, working hours or reported symptoms. Nonetheless, the reduced control sample and its non-random age differences reduce the precision of the morphological comparison and represent a potential source of selection bias that should be borne in mind when interpreting the results. Because we measured no biomarkers of oxidative stress or inflammation, such as malondialdehyde, reduced glutathione, or circulating inflammatory markers, the proposed oxidative mechanism remains unverified in this cohort; establishing it would require a design that pairs exposure assessment with direct biochemical measurement.

## 5 Conclusion

Charcoal-grill restaurant work is associated with a high-particulate occupational environment and with a selective excess of stress-related erythrocyte poikilocytes that persists after adjustment for demographic confounders. The affected forms remained at subclinical frequencies, so the specificity of the pattern, rather than its magnitude, is what points to a mechanical and oxidative effect on the erythrocyte membrane. Given the design and sampling limitations, these findings should motivate larger, longitudinally designed studies with individual-level exposure assessment and biochemical corroboration, alongside practical attention to ventilation and respiratory protection in this occupational setting. Given the restricted scale and exploratory nature of this research, our current conclusions remain tentative and require validation in more robustly powered studies before any causal claims can be made. Accordingly, the associations documented here do not support claims of causality. Consequently, the links documented herein are strictly characterised as correlations rather than direct causal effects, acknowledging that unmeasured confounding variables, such as undocumented tobacco use, may partially account for the observed outcomes.

## Data Availability

The original contributions presented in the study are included in the article/supplementary material, further inquiries can be directed to the corresponding author.
